# The role of chest X-ray in the diagnosis of neonatal respiratory distress syndrome: a systematic review concerning low-resource birth scenarios

**DOI:** 10.1080/16549716.2024.2338633

**Published:** 2024-04-25

**Authors:** Gabriela Silveira Neves, Zilma Silveira Nogueira Reis, Roberta Maia de Castro Romanelli, Jannine dos Santos Nascimento, André Dias Sanglard, James Batchelor

**Affiliations:** a Postgraduate Program in Health Sciences: Child and Adolescent Health – PPGSCA, Faculty of Medicine, Federal University of Minas Gerais, UFMG, Belo Horizonte, Brazil; b Sofia Feldman Hospital, Neonatal Intensive Care Unit, Belo Horizonte, Brazil; c Clinical Informatics and Healthcare Innovation, Faculty of Medicine, University of Southampton, Southampton, UK

**Keywords:** Infant, newborn, radiography, lung, neonatal respiratory distress syndrome, diagnosis

## Abstract

**Background:**

Access to diagnostic tools like chest radiography (CXR) is challenging in resource-limited areas. Despite reduced reliance on CXR due to the need for quick clinical decisions, its usage remains prevalent in the approach to neonatal respiratory distress syndrome (NRDS).

**Objectives:**

To assess CXR’s role in diagnosing and grading NRDS severity compared to current clinical features and laboratory standards.

**Methods:**

A review of studies with NRDS diagnostic criteria was conducted across six databases (MEDLINE, EMBASE, BVS, Scopus-Elsevier, Web of Science, Cochrane) up to 3 March 2023. Independent reviewers selected studies, with discrepancies resolved by a senior reviewer. Data were organised into descriptive tables to highlight the use of CXR and clinical indicators of NRDS.

**Results:**

Out of 1,686 studies screened, 23 were selected, involving a total of 2,245 newborns. All selected studies used CXR to diagnose NRDS, and 21 (91%) applied it to assess disease severity. While seven reports (30%) indicated that CXR is irreplaceable by other diagnostic tools for NRDS diagnosis, 10 studies (43%) found that alternative methods surpassed CXR in several respects, such as severity assessment, monitoring progress, predicting the need for surfactant therapy, foreseeing Continuous Positive Airway Pressure failure, anticipating intubation requirements, and aiding in differential diagnosis.

**Conclusion:**

CXR remains an important diagnostic tool for NRDS. Despite its continued use in scientific reports, the findings suggest that the study’s outcomes may not fully reflect the current global clinical practices, especially in low-resource settings where the early NRDS approach remains a challenge for neonatal survival.

**Trial registration:** PROSPERO number CRD42022336480.

## Paper context

- **Main findings:** Access to diagnostic tools like chest radiography is challenging in resource-limited areas, yet its usage persists in the management of neonatal respiratory distress syndrome despite a decreased dependency due to the imperative for swift clinical decisions.
- **Added knowledge:** Despite its continued significance in scientific literature, the usage of chest radiography as a diagnostic tool for neonatal respiratory distress syndrome may not entirely reflect current global clinical practices, particularly in low-resource settings where early management of neonatal respiratory distress syndrome poses a challenge for neonatal survival.
- **Global health impact for policy and action:** The results underscore the necessity of guidelines for the utilisation of chest radiography to minimise unnecessary ionising radiation exposure while ensuring timely access to critical clinical information for appropriate newborn care.

## Introduction

Neonatal respiratory distress syndrome (NRDS) is a common neonatal disease and the leading cause of death in children worldwide, accounting for approximately 16% of all deaths below five years of age and 35% of deaths among newborns [1]. Socioeconomic status is an important health determinant across maternal and child health outcomes [2] and the majority of neonatal deaths occur in low- and middle-income countries (LMIC) [3]. NRDS is caused by the immature lung structure and function. The lack of pulmonary surfactant, due to either inadequate production or surfactant inactivation in the context of immature lungs, affects the gas exchange leading to acidosis and hypoxemia [4]. The natural course of NRDS is the onset of symptoms at the time of birth with progressive hypoxia and respiratory failure if not treated in time. Therefore, prompt diagnosis is required to ensure an effective treatment and reduce neonatal death rate [5].

Since the definition of NRDS is inaccurate, the current diagnostic includes the assessment of medical records for perinatal risk factors identification, clinical symptoms, radiographic findings, and blood gas analysis with evidence of hypercapnia and hypoxemia [6]. The clinical presentation consists of respiratory symptoms with increased work of breathing, including tachypnea, nasal flaring, grunting, retractions and use of accessory muscles, cyanosis, with decreased air entry on auscultation. The pathognomonic findings on radiography include homogeneous lung disease with diffuse atelectasis, a ground-glass reticulo-granular appearance, with air bronchograms and low lung volumes [7].

In the management of neonatal lung diseases that require NICU admission, chest x-ray (CXR) is the most used medical imaging for the initial diagnosis of major clinical changes in the respiratory profile and is the standard procedure to determine the placement of probes, tubes and catheters [8]. However, social inequalities between high-income countries (HIC) and LMIC are worrying in terms of health and well-being. Lack of access to high-cost technologies and professionals trained to perform diagnostic imaging is part of the challenge in offering due care for preterm newborns.

Equal access to healthcare ensures timely and effective diagnoses, facilitating appropriate care, such as allowing adequate time for transferring newborns to referral centres. While there’s a trend in clinical practice, as reported in guidelines, to decrease CXR use, it often involves substituting with even less accessible exams for low-resource populations. Disparities contribute to the increasing global burden of disease and mortality, with infant mortality in the first day of life being 30 times higher in LMIC [9,10].

Furthermore, evidence supports that ionising radiation causes cellular damage, and that there is a linear increase in lifetime cancer risk, even at low doses of exposure. Neonatal organs which are not fully developed and are more sensitive to CXR, repeated examinations can cause and amplify radiation damage. The risk of the effects of ionising radiation is higher the younger the child is, thus dose reduction is a goal in the field of neonatology [5].

Clinical guidelines aim to minimise exposure to ionising radiation, furthermore CXR is not always available in low-income settings. However, no review has demonstrated whether radiography is necessary for confirming diagnosis [11]. Investigating the importance of CXR in assessing and diagnosing NRDS could improve treatment in resource-limited facilities. Clarifying the need for CXR versus the sufficiency of clinical features could guide future approaches. Identification of the purpose of the CRX in the diagnosis of NRDS should be evaluated as mandatory use, in conjunction with other criteria, for differential diagnosis, to classify the severity of NRDS, to guide treatment or for other reasons.

Therefore, the review aims to determine the necessity of CXR for diagnosing and classifying the severity of NRDS compared to clinical features and laboratory standards.

## Methods

### Eligibility criteria

The systematic review had the International Prospective Registry of Systematic Reviews under PROSPERO number: CRD42022336480. The research protocol followed the recommendations of the PRISMA Statement [12]. To structure the research question about the role of CXR in diagnosing and classifying the severity of NRDS, the acronym PECOS was used. Therefore, in the search for evidence, infants, newborns were considered for (P) Population; for (E) Exposure the CXR; as (C) Comparator the standards of clinical features to establish or assist in the diagnosis of NRDS. Current clinical features, such as evaluation of signs and symptoms, as well as laboratory tests, cited as supporting the diagnosis; as (O) Outcomes the NRDS diagnosis (primary) and NRDS severity classification (secondary); and (S) Study the observational and interventional studies. This research employed two independent pairs of reviewers and a third senior investigator to resolve any discrepancies at each step throughout the entire process.

Studies based on the newborn population with defined criteria for diagnosing NRDS, from the earliest record to the 3rd of March 2023, were included. The language was restricted to English, Portuguese, Spanish, and French. It was considered studies investigating the criteria used to diagnose NRDS and the mandatory use or not of the CXR.

Studies that did not refer to research questions, in addition to incomplete articles, abstracts, review articles, editorials, books, scholar papers, dissertations and theses were excluded.

### Information sources and search strategy

The search was conducted on PubMed (MEDLINE), EMBASE, BVS, Scopus-Elsevier, Web of Science, and Cochrane. Searching process was conducted through descriptors and correlates found in the Medical Subject Heading (MeSH) and descriptors in Health Sciences (DeCS), according to the search strategy of each database.

Complete search strategy, adopting specific descriptors linked to Boolean operators, was (‘Infant, Newborn’ OR Neonate OR Newborn OR ‘Newborn Infant’) AND (Radiography OR ‘Diagnostic X Ray’ OR “Diagnostic X Ray Radiology’’ OR ‘Diagnostic X-Ray’ OR ‘Diagnostic X-Ray Radiology’ OR ‘Radiology, Diagnostic X Ray’ OR ‘X Ray Radiology, Diagnostic’ OR ‘X Ray, Diagnostic’ OR ‘X-Ray Radiology, Diagnostic’ OR ‘X-Ray, Diagnostic’ OR ‘X-Rays, Diagnostic’) AND (Lung OR Chest) AND (‘Respiratory Distress Syndrome, Newborn’ OR ‘Hyaline Membrane Disease’ OR ‘Neonatal Respiratory Distress Syndrome’ OR ‘Disease, Hyaline Membrane’). Whenever possible, the following filters were used: type of studies: only in humans; and methodological design: clinical trials, cohort, and clinical practice guidelines; limited to medical and health subject area; limited to thorax Radiography. Supplementary file 1 provides the full line by line search strategy as run in each database with the sequence of terms that were used to search on interfaces.

**Figure 1.**
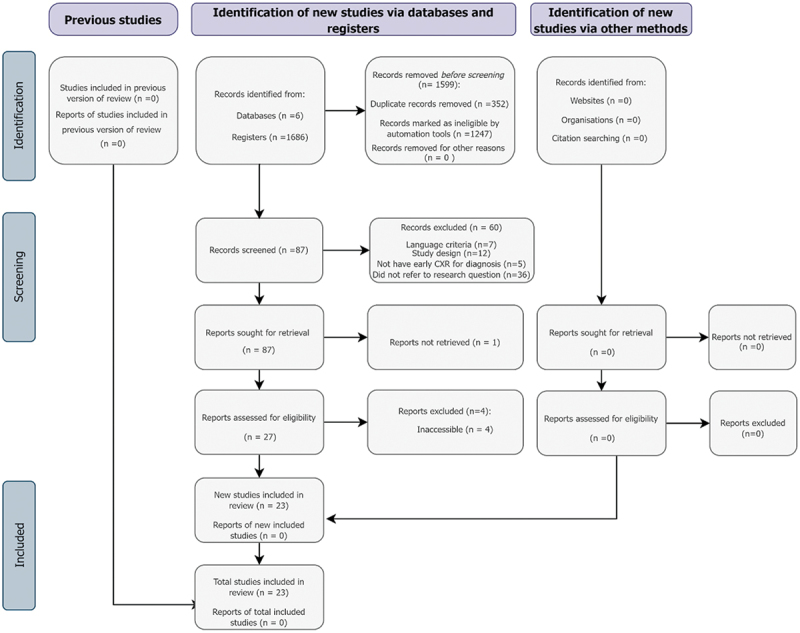
Flowchart with detailed research data for the identified studies for each phase, according to the Preferred Reporting Items for Systematic Reviews and Meta-analyses (PRISMA) [12].

The data search, screening and inclusion procedures are illustrated in Figure 1. In the first phase of the search, 1,686 studies were retrieved. Among these, 762 were sourced from the PubMed database, 635 from Scopus, 1 from Web of Science, 25 from Cochrane, 42 from BVS, and 221 from Embase. After a comprehensive analysis, 23 studies out of 1686 were chosen, involving a total of 2,245 newborns.

### Selection and data collection process

References retrieved from search strategies were exported to StArt _®_ (v.3.3. Beta 03) file [13], and duplicates were removed. Following this procedure, studies were screened based on titles and abstracts, and subsequently, in their full-text versions, according to the inclusion criteria outlined above.

The final selection of included studies was carried out for qualitative and quantitative analysis. Subsequently, data were extracted and the characteristics of the included studies were broken down: authors, year of publication, study period, country, study design, population characteristics, main objective, clues for diagnosis with clinical evaluation, such as oximetry, frequency and signs of respiratory effort, or by laboratory tests and CXR. Any other data of interest that reply to the scientific question was taken into account.

### Data items (outcomes)

Investigations into the rate of NRDS and the importance of CXR in the assessment of NRDS were performed in each study. The main use of this exam was marked as 1) mandatory criterion for the NRDS diagnosis conjoining clinical features, 2) to complete the clinical features, but not as a mandatory for diagnosis, 3) to assess other diseases (differential diagnosis of pulmonary conditions), 4) to classify the severity of NRDS, 5) guide the surfactant administration, or 6) for any other reason, such as verifying the correct placement of devices as an endotracheal tube. The main patterns in the CXR findings to characterise the NRDS were described as well as the criteria considered for differential diagnosis. When available, the time when the CXR was taken was presented. In studies with a control group, the best diagnostic accuracy was described. When classifying the severity of NRDS, the classification method/system was detailed. When used to guide exogenous surfactant replacement, the timing and patterns observed on CXR were revealed.

### Study risk of bias assessment

For the risk of bias in randomised trials, the revised RoB 2.0 tool was used. The methodological quality of selected observational studies was evaluated by the Newcastle-Ottawa Scale (NOS) adjusted for the context of the review [14], detailed in Supplementary file 2.

### Synthesis methods

The primary endpoint of the study was the diagnosis of NRDS, and the effect measures were the number of studies that did or did not recommend CXR as a diagnostic criterion for NRDS. Furthermore, the synthesis of the diagnostic of NRDS with and without CXR support was compared. The secondary outcome was the utilisation of CXR as a criterion for NRDS severity classification. In addition, elements of CXR analysis considered relevant for such classification were extracted and a summary of the topic was provided. Differences in diagnostic rates between CXR and other diagnostic methods, such as clinical features, were summarised, as well as divergences in severity rating rates.

After extraction, the data was summarised in tables. Characteristics of the studies, epidemiological characteristics of the participants, year, author, and outcomes were identified and described. Subgroups of analysis were planned, when available, on the basis of socioeconomic inequalities (LMIC vs. HIC); grades of prematurity (extremely preterm vs. very preterm vs. moderate to late preterm); birth weight categories (low birth weight vs normal birth weight vs high birth weight); and arrangements considering the date of publication.

## Results

The general characterisation of eligible articles is presented in Table 1. Among the 23 articles included, the publication years ranged from 1987 to 2022. The study designs varied, with 9 (39%) being cohort studies, 5 (22%) case-control studies, 8 (35%) cross-sectional studies, and 1 (4%) clinical trial. The sample sizes in these studies ranged from 33 to 235 newborns. Regarding the target population, there were variations among the studies based on the gestational age included.

**Table 1.** General characterisation of eligible articles.

Author/year	Income group	Study design	N	Range of birth weight (grams)	Range of GA (weeks)	Target population	Groups of study
Wood, B.P., et al., [15]	HIC	Clinical trial	60	-	25 to 29	Newborns at risk for NRDS	Endotracheal surfactant vs. placebo (NaCl)
Kurl, S., et al., [16]	HIC	Cross-sectional	99	Median: 3000	24 to 41	Newborns admitted to NICU with early respiratory distress	Clinicians diagnosis vs. radiologists diagnosis
Bober, K., et al., [17]	HIC	Cross- sectional	131	Mean ± DP (min to max): 1898 ± 864 (500 to 4400)	Mean ± DP (min to max): 32 ± 4.4 (24 to 42)	Newborns admitted to NICU with signs of respiratory failure	US vs. CXR for NRDS severity classification
Shahramian, I., et al., [18]	LMIC	Case- control	130	Mean ± DP: 2575 ± 791	Mean ± DP: 35.500 ± 2.213	Newborns admitted to NICU with Apgar score more than 7	Preterm newborn vs. full-term newborns
Raimondi, F., et al., [19]	HIC	Cross- sectional	54	Mean ± DP:1703 ± 583	Mean ± DP: 32.5 ± 2.6	Preterm newborns admitted to NICU with moderate respiratory distress and treated with nCPAP	US vs. CXR for NRDS severity classification
Yin, X., et al., 2014 [20]	UMIC	Case- control	83	Mean ± DP: 2945.3 ± 193.3 (NRDS) and 2969.2 ± 247.3 (control)	Mean ± DP: 38.0 ± 0.7(NRDS) and 38.2 ± 0.8(control)	Full-term newborns with NRDS	NRDS vs. health newborns
Tagliaferro, T., et al., [21]	HIC	Cohort	235	Median: 713 CPAP failure and 805 CPAP success	Median (IQR): 26 (25–26) CPAP failure and 27 CPAP success	All inborn ELBW newborns admitted to NICU	Newborns who succeeded nCPAP vs. newborns who failed nCPAP
El-Malah, H.E. et al., [22]	LMIC	Cohort	100	Mean (min to max): 2400 (2100 to 3000)	≥36 (mean 37.86)	Newborn with clinical and radiographic signs of NRDS.	US vs. CXR for NRDS severity classification
Liu, J., et al., [23]	UMIC	Case- control	100	Mean ± DP: 2320 ± 353 (NRDS) and 2297 ± 411 (control)	Mean ± DP: 34.9 ± 2.7 (NRDS) and 35.1 ± 2.8 (controls)	Newborns admitted to NICU	NRDS vs. health newborns
Sawires, H.K., et al., [24]	LMIC	Case- control	130	Mean ± DP: 1384.22 ± 176.46 (group) and 1580.6 ± 204.44 (control)	Mean ± DP: 29.91± 1.33 (group) and 34.22 ± 1.05 (control)	Preterm newborns admitted to NICU	US vs. CXR for NRDS complications
Abdelsadek, A., et al., [25]	LMIC	Cross- sectional	40	Mean (min to max): 1600 (1000 to 2500)	Mean (min to max) 33 (29 to 35)	Preterm newborns admitted to NICU with respiratory failure within 6h of life and birth weight appropriate for GA	NRDS severity classification
Rachuri, H., et al., [26]	LMIC	Cross- sectional	94	Mean ± DP: 1987 ± 669 (group) and 2349 ± 653 (control)	Mean ± DP: 34.5 ± 3.2 (group) and 35.9 ± 2.7 (control)	Newborns admitted to NICU with respiratory distress who had CXR and US within 4h admission	US vs. Gold Standard (clinic-radiological diagnosis)
Perri, A.; et al., [27]	HIC	Cohort	56	Mean ± DP: 1442 ± 520	Mean ± DP: 31 ± 3	Newborn admitted to NICU with respiratory failure within 2h of life and treated with nCPAP.	US vs. CXR to predict surfactant administration
Li, Y.; Lin, L.; Wang, Q., [28]	UMIC	Cross- sectional	150	Mean ± DP: 1: 2120 ± 430 2: 2050 ± 390 3: 2100 ± 460 4: 2010 ± 470	Mean ± DP: 1: 33.75 ± 1.53 2: 32.78 ± 2.31 3: 33.09 ± 1.67 4: 33.41 ± 1.61	Preterm newborn with LBW and NRDS who underwent alveolar lavage therapy	Expression levels of caspase-3 and Bcl-2 vc. CXR in NRDS severity classification
Grimaldi, C., et al., [29]	HIC	Cohort	52	Mean ± DP: 2065 ± 1174	Median (range): 33 (25–41)	Newborns who needed a CXR because of respiratory conditions during the first 24 h of life	US vs. CXR for NRDS diagnosis
Pasic, I.S., et al., [30]	UMIC	Cross- sectional	51	Median (min to max): 1790 (1570 to 2160)	Median: 31	Newborns ≤35 weeks of GA with positive clinical and laboratory signs of impaired respiratory function	US vs. CXR for NRDS diagnosis
Vardar, G., et al., [31]	UMIC	Cohort	45	Median (IQR): 1290 (975–1720)	Median (IQR): 30 (27–32)	Preterm infants < 34 weeks GA with NRDS	US vs. CXR for NRDS severity classification and to predict surfactant
Kayki, G., et al., [32]	UMIC	Cohort	71	Mean ± DP: 1265 ± 415	Mean ± DP: 29.6 ± 2.3	Preterm newborn admitted to NICU with ≤ 32 weeks GA, treated with nCPAP due to respiratory distress.	US vs. CXR for surfactant administration
Aldecoa-Bilbao, V.; et al., [33]	HIC	Cohort	144	Mean ± DP: 1175 ± 314 (No surfactant) and 1066 ± 397 (Surfactant)	23+0 to 31+6	Preterm newborn admitted to NICU with GA between 23+0 and 31+6 who required noninvasive ventilation for NRDS.	US vs. CXR for surfactant administration
Oktem, A., et al., [34]	UMIC	Cohort	40	Mean ± DP (min to max): 1817.12 ± 211 (600 to 3260)	Mean (min to max): 32 weeks ± 4 days (26 +4 to 37)	Preterm newborns admitted to NICU with NRDS and who need surfactant administration	Before vs. after surfactant administration (US vs CXR)
Reza, M.; et al., [35]	LMIC	Cross- sectional	33	Mean: 1230	Mean (min to max): 29.64 (<28 to 34)	Preterm newborns admitted to NICU	US vs. CXR for NRDS diagnosis
Eldeen, S.M.; et al., [36]	HIC	Case- control	177	Mean ± DP: 2800 ± 580	Mean ± DP: 36.8 ±1.65	Newborns ≥ 35 weeks with NRDS and who required any type of respiratory support	<35 weeks newborns with NRDS vs. ≥ 35 weeks newborns with NRDS
Xiao, Y., et al., [37]	UMIC	Cohort	170	Mean ± DP: 1- mild: 2709 ± 124 2- severe: 1660 ± 750	Mean ± DP: 1- mild NRDS: 34.3 ± 5.14 2- severe NRDS: 30.2 ± 3.39	Newborns admitted to NICU with NRDS	Severe NRDS vs. mild NRDS

HIC: High income country. UMIC: Upper middle-income country. LMIC: Lower middle-income country. nCPAP: Nasal continuous positive airway pressure. NRDS: Neonatal Respiratory distress syndrome. GA: Gestational age. NICU: Neonatal intensive care unit. CXR: Chest x-ray. LBW: Low birth weight. ELBW: Extremely low birth weight. US: Ultrasound.

Table 2 provides an overview of the characterisation of CXR usage and the clinical features associated with NRDS. It’s worth noting that there was no consensus regarding the exact timing of CXR exposure in the included studies. While all studies reported the use of the first CXR for diagnosing NRDS, the timing of this CXR varied. Specifically, in 12 (52%) of the studies, the CXR was conducted between 2 and 24 hours of life. Four (17%) did not define a specific timing, four (17%) considered the timing after admission to the NICU, two (9%) specified the CXR being conducted 2 hours after CPAP initiation, and one (4%) reported that the CXR was done before surfactant administration.

**Table 2.** Characterisation of CXR use and clinical features associated with NRDS.

Author/year	Time of the first CXR	CXR features associated to NRDS	Clinical features associated to NRDS	Protocol/guideline for NRDS diagnosis	NRDS severity classification	Other use for CXR	Summary of the topic
Wood, B.P., et al., [15]	Within 90 minutes of NICU admission	Reduced lung expansion, reticular densities, air bronchogram, consolidation of lungs.	Mean airway pressure and FiO_2_ required for adequate ventilation	No	Class 1 to 3, proposed by the authors	Differential diagnosis and surveillance	The radiological data provided support of the surfactant replacement efficacy evaluation.
Kurl, S., et al., [16]	Within first 3 h of life	Homogeneous, opaque infiltration, air bronchogram.	Retractions, tachypnea, supplemental oxygen, acid-base disturbance in arterial blood gas analysis	No	Mild or Moderate (clinical definition)	Differential diagnosis	There was 95% agreement between clinicians and radiologists for NRDS diagnosis. The first CXR taken had the greatest impact in the care of neonates.
Bober, K., et al., [17]	Within first 24 h of life	Not described but based on Hansen T, Corbet A (1991) criteria	CRIB	Hansen and Corbet; 1991	Grade 1 to 4	None	US examination cannot replace CXR as it overestimates the diagnosis of NRDS
Shahramian, I., et al., [18]	Undefined	Granularity of lungs, air bronchogram and white out lungs with loss of cardiac borders	None	No	Normal, Mild, moderate and severe	None	Increase of serum Brain Natriuretic Peptide (BNP) level correlates to NRDS severity (CXR) in premature infants.
Raimondi, F., et al., 2014 [19]	After 120 minutes from the initiation of CPAP	Ground glass shadowing, air bronchograms, alveolar shadowing, white-out of the lung fields with obscuring of the cardiac border	Retractions, nares dilatation, grunting and acid-base disturbance in arterial blood gas analysis	No	Grade 1 to 4 by Arthur (2001)	Surveillance and prediction of CPAP failure	After a 2-hour nCAP trial, US largely outperformed CXR in predicting the need for intubation.
Yin, X., et al., [20]	Undefined	Bilateral transmittance reduced, small mesh-like particle shadow, air bronchogram; blurred heart and diaphragm contours, white lung	Moaning, dyspnea, acid-base disturbance in arterial blood gas analysis, supplemental oxygen and mechanical ventilation	Shao et al., 2011	Grade I to IV by Shao et al., 2011	None	SP-B expressed in bronchoalveolar lavage fluid is inversely related to the severity of NRDS (CXR) in full-term newborn.
Tagliaferro, T., et al., [21]	Within first 2h of life	Reticulogranular infiltrates, air bronchogram, alveolar opacification making the borders of the heart, thymus and diaphragm unclear, lungs quite airless	Acid-base disturbance in arterial blood gas analysis	Kero and, Mäkinen, 1979	Class 1 to 3 by Kero and Mäkinen (1979)	Prediction of CPAP failure	Early radiologic evidence of severe NRDS is predictive of CPAP failure, especially in infants with GA≤26 weeks
El-Malah, H.E. et al., [22]	Within first 24 h of life	Reticulogranular or ground glass opacification, progressive hypo-aeration and air bronchograms.	CRIB	Hansen and Corbet; 1991	Stage I to IV by Hansen T, Corbet A (1991)	Follow up treatment of NRDS	The US can be an alternative diagnostic imaging modality for CXR in follow up treatment of NRDS and subsequent reduction dose of radiation
Liu, J., et al., [23]	After NICU admission	Hypoexpansion, fine granular densities, air bronchogram, ground-glass opacities, blurred cardiac borders, or white lungs	Tachypnea, grunting, retractions, cyanosis, reduced breath sounds, ventilatory support, acid-base disturbance in arterial blood gas analysis	No	Grade 1 to 4 by Ayachi et al. (2005), Bouziri et al. (2007), Faix et al. (1989) and Liu et al. (2010)	None	The US is accurate and reliable to diagnose NRDS. More research is needed to replace CXR.
Sawires, H.K., et al., [24]	Within first 6h of life	Ground glass veiling, bilateral reticulonodular pattern, air bronchogram, bilateral symmetric parenchymal opaqueness	Tachypnea, dyspnea, retraction, grunting or cyanosis, acid-base disturbance in arterial blood gas analysis	No	Grade 1 to 4	Differential diagnosis, surveillance and detection of NRDS complications	US is superior to CXR in the detection of NRDS complications (except for pneumothorax). It could be value in reducing exposure to unnecessary radiation
Abdelsadek, A., et al., [25]	Within first 6 h of life	Hypovolemic lung reticulogranular mottling or without air bronchograms, bilateral opacification of lungs	Down score and laboratory analysis	No	Mild or Severe NRDS	None	US cannot replace standard CXR in diagnosing potential causes of neonatal respiratory failure because of its tendency to over-diagnose NRDS
Rachuri, H., et al., [26]	Within 4 h of NICU admission	Diffuse atelectasis,‘ground glass’ appearance of the lung fields, low lung volume, air bronchograms	Tachypnea, retractions and/or grunting, Downe’s score	No	No	Differential diagnosis and detection of NRDS complications	US can be used to diagnose different etiologies of respiratory distress in neonates. However, CXR is superior to evaluate complications of NRDS.
Perri, A.; et al., [27]	Within 2h from the initiation of CPAP (before surfactant administration)	Reduced radiolucency, air bronchogram, blurred cardiac and diaphragmatic margins, white lung fields	Shallow breathing, grunting, retractions, acid-base disturbance in arterial blood gas analysis	European Consensus Guidelines, 2016	Class 1 to 4 by Cattarossi et al. (2010)	To guide surfactant administration	US predicts the need for surfactant more reliably than CXR
Li, Y.; Lin, L.; Wang, Q., [28]	Within first 4 h of life	Reduced radiolucency, air bronchogram, unclear heart and diaphragmatic surfaces, white lung syndrome	Dyspnea, grunts, and acid-base disturbance in arterial blood gas analysis	Shao et al., 2011	Grades 1 to 4	None	The severity of NRDS in the CXR is positively related with the concentration of caspase-3 in alveolar lavage fluid, and negatively correlated with the expression level of Bcl-2
Grimaldi, C., et al., [29]	Within first 24 h of life	Ground glass shadowing, air bronchograms, confluent alveolar shadowing, and complete white lungs obscuring the cardiac border	Dyspnea, cyanosis, retraction, respiratory support, surfactant therapy	No	Grades 1 to 4 by Agrons et al. (2005), Lobo (2006)	Differential diagnosis and detection of complications of NRDS	The US is superior to CXR for NRDS diagnosis, for differential diagnosis and complications. However, CXR remains necessary for newborns in mechanical ventilation.
Pasic, I.S., et al., [30]	Undefined	Fine homogenous, ground-glass shadowing, air bronchogram, alveolar shadowing obscuring cardiac border	Not clear: clinical signs and acid-base disturbance in arterial blood gas analysis	No	Stage I to IV by Drorbaugh and Fogg (1956)	None	US can be used as complementary modality to CXR, decreasing the number of ionizing radiations in premature newborns
Vardar, G., et al., [31]	Within first 2 h of life	Reticulogranular or ground-glass pattern, air bronchograms, bilateral opacification of lungs with loss of cardiac borders	Not clear: clinical signs, acid-base disturbance in arterial blood gas analysis, FiO2	European Consensus Guidelines, 2016	Stage I to IV by Wambach and Hamvas (2015)	To guide surfactant administration and predict CPAP failure	US is superior to CXR to predict severity of NRDS, surfactant administration and CPAP failure
Kayki, G., et al., [32]	Within first 2 h of life	Reticular appearance, decreased transparency, air bronchogram, white lung with obscuring cardiac border	Not clear: clinical signs and acid-base disturbance in arterial blood gas analysis	European Consensus Guidelines, 2019	Stage I to IV by Perri et al. (2018), Arthur (2001)	To guide surfactant administration	Earlier US (20–30 min) is superior to CXR to predict surfactant administration
Aldecoa-Bilbao, V.; et al., [33]	After NICU admission	Decreased pulmonary expansion, generalized reticulogranular lung opacities and air bronchograms	Shallow breathing, tachypnea, grunting, nasal flaring, and retractions	No	CXR score (0–8 points) adapted from Perri et al. (2018)	To guide surfactant administration	US showed higher sensitivity and predictive values compared with CXR to predict surfactant administration.
Oktem, A., et al., [34]	Before Surfactant administration	Granular pattern of lung parenchyma, air bronchograms and atelectasis	tachypnea, nasal flaring, retractions, or grunting	No	No	Surveillance, to guide surfactant administration, and differential diagnosis	US is superior to CXR in differential diagnosis, and can be used for surveillance without risks of ionization
Reza, M.; et al., [35]	Within first 24 h of life	Lung consolidation, air bronchogram, white-lung appearance.	None	No	According to grades 1 to 4	None	There was an agreement of 63.3% between US and CXR in NRDS diagnosis.
Eldeen, S.M.; et al., [36]	Undefined	Ground glass, air bronchogram, low lung volume	Tachypnea, apnea, reduced breath sounds, cyanosis, surfactant therapy	Local guideline	Mild to severe by Shashidha (2016), Hansen (1991)	None	NRDS among full-term and near-term newborns present mostly mild-to-moderate courses.
Xiao, Y., et al., [37]	Within first 6 h of life	Reticular shadows, white-out appearance, heart shape blurred	None	No	Grade I to IV by Gómez (2020), Hiroyuki (2018)	To guide surfactant, surveillance	US and CXR had the same diagnostic effect on NRDS

CRIB: clinical risk index for babies Score. US: Ultrasound. CPAP: continuous positive airway pressure. CXR: chest x-ray. NRDS: neonatal respiratory distress syndrome.

### Primary endpoint

Despite the differing objectives of each included study, they consistently utilised CXR as a reference for diagnosing NRDS. In summary, some reports emphasised that CXR cannot be replaced by other tools for diagnosing NRDS [17,22,23,25,26,29,30]. Furthermore, the first CXR taken had the greatest impact on neonatal care [16,21]. There was a consensus among health professionals in diagnosing with the exam, including agreement between clinicians and radiologists [16]. Additionally, there was agreement between CXR and other exams, such as ultrasound (US), for NRDS diagnosis [35,37]. However, while CXR has the ability to support the prediction of surfactant administration [15], it can be replaced by other tools for this purpose [27,31–34].

### Secondary endpoint

The importance of CXR in classifying the severity of NRDS was emphasised in 21 (91%) of the studies. The classification systems varied, typically consisting of either three or four classes, often referred to as stages or grades. The main characteristics observed on CXR progressively worsen with higher NRDS severity classifications. These principal patterns include a fine ground glass appearance with reduced lung volume and an air-bronchogram within the cardiac shadow. Selected studies reported agreement between CXR and other exams (e.g. the US) for NRDS severity classification [18,20,28], while others suggested that CXR was surpassed by alternative methods [31].

In terms of the standards of clinical features for establishing or assisting the diagnosis of NRDS, three articles (13%) did not consider it at all. Additionally, CXR had other applications in the included studies, including 6 (26%) for differential diagnosis, 6 (26%) for surveillance and follow-up treatment, 6 (26%) to guide surfactant administration, 3 (13%) to predict CPAP failure, and 3 (13%) to detect complications of NRDS. To support the diagnostic decision, 9 (39%) studies referred to a protocol or guideline, while 14 (61%) did not mention any specific guidelines or protocols. When compared, other diagnostic tools were superior to CXR in various roles, including predicting CPAP failure [31], predicting the need for intubation [19], making a differential diagnosis [34], and for NRDS surveillance [34].

The quality of the selected studies was assessed using the NOS Scale, with a full description provided in the supplementary file 3, and the RoB 2 tool. The RoB 2.0 tool was employed to assess the risk of bias in the randomised trial, with the following judgements for each domain: (1) Randomisation process: Some concerns; (2) Deviations from intended interventions: Some concerns; (3) Missing outcome data: Low risk; (4) Measurement of the outcome: High risk; (5) Selection of the reported result: Low risk; (6) Overall Bias: High risk of bias.

Overall, the majority of studies were of good to high quality, with 9 classified as high quality, 9 as good quality, according to NOS, and 2 rated as poor quality according to NOS and ROB2 each. A causal inference is constrained by risk of bias in some studies, the main concerns being the lack of adjustment for key potential confounders such as gestational age and birth weight [16,19,25,27,31,34,35]; assessment of outcome due to an inappropriate or not-described statistical approach for comparing NDRS diagnoses techniques (CXR and other) [16,17,25]; the lack of representativeness of the NRDS cases [18,20,23,24]; or lack of independent blind assessment (e.g diagnosis based on CXR and medical records by independent professionals or diagnosis based on CXR blinded to the researcher [21,25,32,34,37].

## Discussion

This review evaluated the importance of CXR for the diagnosis and classification of NRDS severity. Among the 23 studies included, all reported CXR as a standard diagnostic tool. Additionally, 21 studies used it to classify NRDS severity. There were other uses related to imaging as well: six studies for differential diagnosis, six for surveillance, six to guide surfactant administration, three to detect NRDS complications, and three to predict CPAP failure. It’s important to interpret these findings with caution since CXR was one of the inclusion criteria for this review.

Early diagnosis of NRDS, necessary to anticipate therapeutic measures, depends on a combination of clinical signs and symptoms, laboratory analyses, and CXR [38]. While CXR has traditionally been considered the standard diagnostic tool for NRDS, in clinical practice, it may not be as useful for making the final diagnosis in certain circumstances. For instance, in cases of congenital pneumonia and severe NRDS, where similarities are found in CXR findings [17,25]. Moreover, the guidelines recommend making a decision on surfactant administration based on clinical features, irrespective of CXR results [39]. Furthermore, in situations where CXR is not feasible, especially in resource-constrained environments or to minimise ionising radiation exposure, clinical classification of severity may serve as an alternative, as it demonstrates correlation with radiological findings [38]. This review did not encompass scenarios with limited resources, considering the socio-economic classification of the majority of selected articles. Future studies focused on obtaining answers in LMIC scenarios may provide specific evidence on this issue.

Chronologically, early studies demonstrated the role of CXR in classifying the severity and prognosis of NRDS, which aided in identifying infants requiring surfactant administration. It also facilitated treatment surveillance, allowing assessment before and after surfactant administration [15]. However, a significant development in neonatology, particularly the early use of nasal CPAP since the 1990s, led to a shift in NRDS severity classification towards clinical determination [40]. This change has resulted in reduced reliance on mechanical ventilation and surfactant use [39].

Among the selected studies, the significance of the earliest CXR in the care of newborns was evident. It demonstrated the ability to detect most lung diseases in the first hours of life [16]. At one point, conducting an initial CXR was deemed a standard practice for diagnosing NRDS and for surveillance, particularly in extremely premature infants [15]. Additionally, it was considered essential for differentiating respiratory disorders in newborns and for precise placement of catheters, probes, and endotracheal tubes [17]. Follow-up images also served to monitor therapeutic effects and reduce morbidities like bronchopulmonary dysplasia (BPD) by minimising mechanical ventilation [38]. However, repeated examinations posed risks to neonates due to ionising radiation exposure [17]. As a result, researchers explored alternative techniques to replace CXR due to these risks. Three studies compared CXR with laboratory tests, including expression levels of cysteine aspartic protease-3 (capase-3) and B-cell lymphoma gene-2 (Bcl-2) [28], levels of brain natriuretic peptide (BNP) [18], and surfactant protein B (SP-B) expression [20], while 16 studies focused on the use of US [17,19,22–27,29–35,37]. While alternative diagnostic methods were investigated to complement or even surpass CXR’s functions, the recommendations for its use began to be questioned over time. Our interpretation of this outcome underscores the enduring importance of clinical features over time, regardless of diagnostic tools.

In summarising the selected articles for this review, several investigations have emphasised the significance of early CXR during the course of neonatal respiratory distress syndrome (NRDS). Kurl et al. (1997) highlighted its impact in detecting critical conditions, such as pneumothoraces, before severe clinical deterioration occurs [16]. Additionally, Bober et al. (2006) found it to be essential for the differential diagnosis of respiratory disorders in neonates [17]. Furthermore, Tagliaferro et al. (2015) explored its potential in predicting CPAP failure within the first 72 hours of life, particularly in ELBW infants. While one study confirmed this potential [21], Raimondi (2014) also demonstrated that a non-ionising examination could potentially replace the need for CXR [19].

### Strength and limitations of the review

The main contribution of this study was to emphasise the evolving use of complementary exams over time and the need to review the role of CXR in clinical practices. Despite technological advancements in neonatology, the CXR associated with clinical features remains the standard reference for diagnosing NRDS.

The results found in this review have limitations, as the studies evaluated did not address the risks and benefits of the systematic use of CXR, nor did they consider the implications of repeated exams for NRDS follow-up. We believe that there is a future agenda to reevaluate recommendations for the mandatory use of CXR whenever NRDS is suspected. Providing guidelines on when to use this tool could be valuable in guiding clinical practice, with the dual aim of minimising unnecessary radiation exposure and ensuring timely access to essential clinical information. Furthermore, although the risk of bias in most studies was low, it’s important to note that the primary objectives of the selected articles did not revolve around comparing clinical and radiological methods for diagnosing NRDS or assessing its severity. Some of these studies aimed to compare CXR with other diagnostic tools, such as the US, for NRDS diagnosis, or to predict the use of surfactant, among other objectives. The significant variation in study objectives was a limiting factor in interpreting the results for clinical practice.

## Conclusion

The role of CXR has evolved over time, from NRDS diagnosis and severity classification to differential diagnosis and surfactant treatment surveillance. Still, CXR is considered a standard tool for confirmatory NRDS diagnosis. Although new complementary exams to assess NRDS in newborns have been studied over the years, the clinical features kept the importance for establishing or assisting the diagnosis of NRDS.

The scarcity of studies dedicated to assessing the relevance of CXR for NRDS evaluation has left it uncertain whether CXR assessment is mandatory for the diagnosis and severity classification of NRDS. Despite its continued use in scientific reports, the findings suggest that the study’s outcomes may not fully reflect the current global clinical practices, especially in low-resource settings where the early NRDS approach remains a challenge for neonatal survival.

## Supplementary Material

Supplemental Material

## Data Availability

Datasets used or analysed during the current study can be obtained from the corresponding author upon reasonable request.

## Abbreviations

CXR: chest X-ray
NRDS: Neonatal Respiratory Distress Syndrome
CPAP: Continuous Positive Airway Pressure
NICU: Neonatal Intensive Care Unit
LMIC: Lower Middle-income Country
NOS: Newcastle-Ottawa Scale
HIC: High Income Country
UMIC: Upper Middle-income Country
GA: Gestational Age
LBW: Low Birth Weight
ELBW: Extremely Low Birth Weight
US: Ultrasound
