# Supplementary material for: The role of chest X-ray in the diagnosis of neonatal respiratory distress syndrome: a systematic review concerning low-resource birth scenarios
Source: Glob Health Action. 2024 Apr 25;17(1):2338633. doi: 10.1080/16549716.2024.2338633 (PMC11047214; doi:10.1080/16549716.2024.2338633)
Supplement: Supplemental Material [file ZGHA_A_2338633_SM3663.docx]

**SUPPLEMENTARY FILES**

**Supplementary file** **1. The search strategy performed in each database.**

| **Database** | **URL to search strategy** |
| --- | --- |
| PUBMED | ("Infant, Newborn" OR "Infants, Newborn" OR Neonate OR Neonates OR Newborn OR "Newborn Infant" OR "Newborn Infants" OR Newborns) AND ("Respiratory Distress Syndrome, Newborn" OR "Hyaline Membrane Disease" OR "Neonatal Respiratory Distress Syndrome" OR "Disease, Hyaline Membrane" OR "Diseases, Hyaline Membrane" OR "Hyaline Membrane Diseases") AND (Radiography OR "Diagnostic X Ray" OR "Diagnostic X Ray Radiology" OR "Diagnostic X-Ray" OR "Diagnostic X-Ray Radiology" OR "Diagnostic X-Rays" OR "Radiology, Diagnostic X Ray" OR "Radiology, Diagnostic X-Ray" OR "X Ray Radiology, Diagnostic" OR "X Ray, Diagnostic" OR "X-Ray Radiology, Diagnostic" OR "X-Ray, Diagnostic" OR "X-Rays, Diagnostic") AND (Lung OR Chest) |
| SCOPUS | ALL(( ( ( "Diagnostic Imaging" OR radiography OR "Imaging, Diagnostic" OR "Imaging, Medical" OR "Medical Imaging" OR "Diagnostic X Ray" OR "Diagnostic X Ray Radiology" OR "Diagnostic X-Ray" OR "Diagnostic X-Ray Radiology" OR "Diagnostic X-Rays" OR "Radiology, Diagnostic X Ray" OR "Radiology, Diagnostic X-Ray" OR "X Ray Radiology, Diagnostic" OR "X Ray, Diagnostic" OR "X-Ray Radiology, Diagnostic" OR "X-Ray, Diagnostic" OR "X-Rays, Diagnostic" ) AND ( "Lung" ) ) AND ( ( "Respiratory Distress Syndrome, Newborn" OR "Hyaline Membrane Disease" OR "Neonatal Respiratory Distress Syndrome" OR "Disease, Hyaline Membrane" OR "Diseases, Hyaline Membrane" OR "Hyaline Membrane Diseases" ) ) ) AND ( ( "Infant, Newborn" OR "Infants, Newborn" OR neonate OR neonates OR newborn OR "Newborn Infant" OR "Newborn Infants" OR newborns ) ) AND ( LIMIT-TO ( DOCTYPE , "ar" ) ) AND ( LIMIT-TO ( SUBJAREA , "MEDI" ) OR LIMIT-TO ( SUBJAREA , "HEAL" ) ) AND ( LIMIT-TO ( EXACTKEYWORD , "Newborn" ) OR LIMIT-TO ( EXACTKEYWORD , "Thorax Radiography" ) ) AND ( EXCLUDE ( LANGUAGE , "German" ) OR EXCLUDE ( LANGUAGE , "Chinese" ) ) AND ( EXCLUDE ( LANGUAGE , "Dutch" ) ) AND ( EXCLUDE ( LANGUAGE , "Italian" ) OR EXCLUDE ( LANGUAGE , "Russian" ) ) AND ( EXCLUDE ( LANGUAGE , "Polish" ) OR EXCLUDE ( LANGUAGE , "Turkish" ) ) AND ( EXCLUDE ( LANGUAGE , "Czech" ) ) AND ( EXCLUDE ( LANGUAGE , "Swedish" ) ) AND ( EXCLUDE ( LANGUAGE , "Bulgarian" ) OR EXCLUDE ( LANGUAGE , "Croatian" ) OR EXCLUDE ( LANGUAGE , "Danish" ) OR EXCLUDE ( LANGUAGE , "Japanese" ) )) AND NOT ("case report") |
| WEB OF SCIENCE | (((("Infant, Newborn" OR "Infants, Newborn" OR "Neonate" OR "Neonates" OR "Newborn" OR "Newborn Infant" OR "Newborn Infants" OR "Newborns" )) AND TS=(("Respiratory Distress Syndrome, Newborn" OR "Hyaline Membrane Disease" OR "Neonatal Respiratory Distress Syndrome" OR "Disease, Hyaline Membrane" OR "Diseases, Hyaline Membrane" OR "Hyaline Membrane Diseases"))) AND TS=(("Lung" OR "Chest"))) AND TS=(("radiography" OR "x ray")) |
| COCHRANE | ("Infant, Newborn" OR "Infants, Newborn" OR Neonate OR Neonates OR Newborn OR "Newborn Infant" OR "Newborn Infants" OR Newborns) AND ("Respiratory Distress Syndrome, Newborn" OR "Hyaline Membrane Disease" OR "Neonatal Respiratory Distress Syndrome" OR "Disease, Hyaline Membrane" OR "Diseases, Hyaline Membrane" OR "Hyaline Membrane Diseases") AND (Radiography OR "Diagnostic X Ray" OR "Diagnostic X Ray Radiology" OR "Diagnostic X-Ray" OR "Diagnostic X-Ray Radiology" OR "Diagnostic X-Rays" OR "Radiology, Diagnostic X Ray" OR "Radiology, Diagnostic X-Ray" OR "X Ray Radiology, Diagnostic" OR "X Ray, Diagnostic" OR "X-Ray Radiology, Diagnostic" OR "X-Ray, Diagnostic" OR "X-Rays, Diagnostic") AND (Lung OR Chest) |
| EMBASE | (newborn) and ('neonatal respiratory distress syndrome') and (radiography or x ray) and (lung or thorax or chest) |
| BVS | (recém-nascido OR "Infant, Newborn" OR "Recién Nacido" OR nouveau-né OR "Criança Recém-Nascida" OR "Crianças Recém-Nascidas" OR "Lactente Recém-Nascido" OR "Lactentes Recém-Nascidos" OR neonato OR neonatos OR recém-nascidos OR "Infants, Newborn" OR neonate OR neonates OR newborn OR "Newborn Infant" OR "Newborn Infants" OR newborns) AND ("Síndrome do Desconforto Respiratório do Recém-Nascido" OR "Respiratory Distress Syndrome, Newborn" OR "Síndrome de Dificultad Respiratoria del Recién Nacido" OR "Syndrome de détresse respiratoire du nouveau-né" OR "Síndrome da Angústia Respiratória do Recém-Nascido" OR "Síndrome do Desconforto Respiratório em Recém-Nascidos" OR "Doença da Membrana Hialina" OR "Hyaline Membrane Disease" OR "Enfermedad de la Membrana Hialina" OR "Maladie des membranes hyalines" OR "Neonatal Respiratory Distress Syndrome" OR "Disease, Hyaline Membrane" OR "Diseases, Hyaline Membrane" OR "Hyaline Membrane Diseases") AND (radiografia OR radiography OR radiografía OR radiographie OR "Diagnóstico Radiológico por Raios X" OR "Diagnóstico por Raios X" OR "Diagnostic X Ray" OR "Diagnostic X Ray Radiology" OR "Diagnostic X-Ray" OR "Diagnostic X-Ray Radiology" OR "Diagnostic X-Rays" OR "Radiology, Diagnostic X Ray" OR "Radiology, Diagnostic X-Ray" OR "X Ray Radiology, Diagnostic" OR "X Ray, Diagnostic" OR "X-Ray Radiology, Diagnostic" OR "X-Ray, Diagnostic" OR "X-Rays, Diagnostic") AND (pulmão OR lung OR pulmón OR poumon OR tórax OR thorax OR tórax OR thorax) AND ( db:("LILACS" OR "IBECS" OR "BINACIS" OR "CUMED")) |

**Supplementary file** **2. Newcastle-Ottawa Scale (NOS) adjusted for the context of the review.**

NEWCASTLE - OTTAWA QUALITY ASSESSMENT SCALE

(adapted for case-control studies)

**Selection (4 stars)**

1. Is the case definition adequate?
2. ☆ yes, with independent validation (researchers validated NRDS diagnosis)

NRDS diagnosis by chest x-ray, clinical findings or both described on medical records. Cases must be identified by qualified healthcare professionals or based on medical records and confirmed diagnoses. Cases must be properly documented, and medical records or sources of information used to identify cases must be clearly reviewed and recorded.

1. zero: no description (or without independent validation)

2) Representativeness of the cases

a) ☆ consecutive or obviously representative series of cases (consecutive or random choice of cases)

All eligible cases with NRDS diagnosis in the first 72 hours of life, all cases at NICU or sample calculation

b) zero: potential for selection biases or not stated

c) zero: Studies that evaluated specific subgroups of newborns (LBW, GA, diabetes)

3) Selection of Controls

a) ☆ community controls (control NRDS-no, with similar characteristics to NRDS) Control defined by the authors. Examples: newborns without NRDS or different age groups (preterm vs. term) or different birth weight ranges or use of surfactant replacement (yes vs. no) or other diagnostic test (CXR vs. US)

b) zero: Evaluated under different conditions or time

c) zero: no description

4) Definition of Controls

a) ☆ Explicit description (without NRDS and other diseases)

According to the definition of control by the author, the control conditions must be explicit. Example: controls have no report of NRDS or the division of different age groups (premature vs. term) or different birth weights or use of surfactant replacement (yes vs. no) or groups with different tests (CXR vs. US)

b) zero: no description of source

**Comparability (2 stars)**

1) Comparability of cases and controls on the basis of the design or analysis

a) ☆ study controls for NRDS diagnosis (if the control group was NRDS-no)

b) ☆ study controls for any additional factor (when a second factor was match criterion) Examples: groups of age and birth weight, maternal characteristics: hypertension, diabetes, antenatal corticosteroids.

**Exposure (3 stars)**

1) Ascertainment of exposure (CXR): maximum one star

a) ☆ secure record (CXR and clinical findings were obtained in medical charts) OR

b) ☆ structured interview where blind to case/control status (CXR was retrospectively analysed blinded of NRDS diagnosis)

c) zero: written self-report (CXR was analysed after case or control definition)

d) zero: no description

2) Same method of ascertainment for cases and controls

a) ☆ yes (CXR and clinical findings were analysed similarly in case or control newborns)

b) zero: no

3) Non-Response rate

a) ☆ same rate for both groups (Is there any newborn without X-ray or clinical findings?

b) zero: non respondents described

c) zero: rate different and no designation

NEWCASTLE - OTTAWA QUALITY ASSESSMENT SCALE

(adapted for cohort studies)

**Selection (4 stars)**

1) Representativeness of the exposed cohort

a) ☆ truly representative of the average (NRDS diagnosis based on CXR in the community) - population cohort on NRDS.

b) ☆ somewhat representative of the average in the community (multicenter studies on NRDS, random sample of NRDS newborns)

c) zero: selected group of users, sample from selected groups of study, convenience sample (e.g., Preterm, LBW)

d) zero: no description of the derivation of the cohort

2) Selection of the non-exposed cohort

a) ☆ drawn from the same community as the exposed cohort - neonatal sample

 with NRDS diagnosis based on clinical findings (additionally to CXR).

b) zero: drawn from a different source (neonatal sample with NRDS diagnosis based only on CXR)

c) zero: no description of the derivation of the non-exposed cohort

3) Ascertainment of exposure

a) ☆ secure record (CXR findings reported by researcher even based on medical records)

b) zero: structured interview (non-applicable)

c) zero: written self-report (CXR findings reported based on medical records without the researcher scrutiny)

d) zero: no description

4) Demonstration that outcome of interest was not present at start of study

a) ☆ yes (NRDS diagnosis based on validated guidelines)

b) zero: no

**Comparability (2 stars)**

1. Comparability of cohorts on the basis of the design or analysis

a) ☆ study controls for (clinical characteristics: GA, birth weight)

b) ☆ study controls for any additional factor (Maternal characteristics such as hypertension or diabetes)

**Outcome (3 stars)**

1) Assessment of outcome

a) ☆ independent blind assessment - NRDS vs. No-NRDS (Diagnosis based on CXR, medical records, by independent professionals / or diagnosis based on CXR blinded for the researcher/ studies for NRDS severity classification by independent professionals or researchers).

b) ☆ record linkage (unified database according to methodology of data collection)

c) zero: self-report

d) zero: no description

2) Was follow-up long enough for outcomes to occur

a) ☆ yes (proper definition of follow-up to obtain a NRDS diagnosis)

b) zero: no

3) Adequacy of follow up of cohorts

a) ☆ complete follow up - all subjects accounted for diagnosis at 72 hours of life

b) ☆ subjects lost to follow up unlikely to introduce bias - small number lost to follow up < 5%, or description provided of those lost. Death before NRDS diagnosis, missing data.

c) zero: follow up lost > 5% and no description of those lost

d) zero: no statement

NEWCASTLE - OTTAWA QUALITY ASSESSMENT SCALE

(adapted for cross-sectional studies)

**Selection (5 stars)**

1. Representativeness of the sample:
   - 1. ☆ Truly representative of the average in the target population (all subjects or random sampling). All eligible cases with NRDS diagnosis in the first 72 hours of life, all cases at NICU or sample calculation
     2. ☆ Somewhat representative of the average in the target group (non-random sampling).
     3. zero: Selected group of users/convenience sample.
     4. zero: No description of the derivation of the included subjects.
2. Sample size:
   - 1. ☆ Justified and satisfactory (including sample size calculation).
     2. zero: Not justified.
     3. zero: No information provided
3. Non-respondents: study group (CXR vs. US, CXR vs. clinic, NRDS grades)
   - 1. ☆ Proportion of target sample recruited attains pre-specified target or basic summary of non-respondent characteristics in sampling frame recorded.
     2. zero: Unsatisfactory recruitment rate, no summary data on non-respondents.
     3. zero: No information provided
4. Ascertainment of the exposure (risk factor):
   - 1. ☆☆ Medical records/chest x-ray diagnosis/clinical diagnosis
     2. ☆ structured interview where blind to case/control status
     3. zero: Unsatisfactory information

**Comparability (2 stars)**

1. Comparability of subjects in different outcome groups on the basis of design or analysis. Confounding factors controlled.
   - 1. ☆☆ Data/ results adjusted for relevant predictors/risk factors/confounders e.g., age, sex, etc.
     2. Zero: Data/results not adjusted for all relevant confounders/risk factors/information not provided.

**Outcome (3 stars):**

1. Assessment of outcome:
   - 1. ☆☆ independent blind assessment - NRDS vs. No NRDS (CXR, medical records, US, etc.)
     2. ☆☆ Unblinded assessment using objective validated methods.
     3. ☆☆Used non-standard or non-validated methods with gold standard.
     4. Zero: No description
2. Statistical test:
   - 1. ☆ Statistical test used to analyse the data clearly described, appropriate and measures of association presented including confidence intervals and probability level (p value).
     2. Zero: Statistical test not appropriate, not described or incomplete.

**Supplementary file 3.** **Quality assessment of selected studies based on the Newcastle-Ottawa Scale**

| **Author / year** | **Study design** | **Selection** | **Comparability** | **Exposure/ Outcome** | **Total** |
| --- | --- | --- | --- | --- | --- |
| Kurl, S., et al., 1997 [17] | Cross-sectional | ☆☆☆ | - | ☆ | 4/10 |
| Bober, K., et al., 2006 [18] | Cross-sectional | ☆☆☆☆ | ☆ | ☆ | 6/10 |
| Shahramian, I., et al., 2013 [19] | Case-control | ☆ | ☆☆ | ☆☆☆ | 6/9 |
| Raimondi, F., et al., 2014 [20] | Cross-sectional | ☆☆☆☆☆ | - | ☆☆ | 7/10 |
| Yin, X., et al., 2014 [21] | Case-control | ☆☆☆ | ☆☆ | ☆☆☆ | 8/9 |
| Tagliaferro, T., et al., 2015 [22] | Cohort | ☆☆☆☆ | ☆☆ | ☆☆ | 8/9 |
| El-Malah, H.E. et al., 2015 [23] | Cohort | ☆☆☆ | ☆ | ☆☆☆ | 7/9 |
| Liu, J., et al., 2015 [24] | Case-control | ☆☆☆ | ☆☆ | ☆☆☆ | 8/9 |
| Sawires, H.K., et al., 2015 [25] | Case-control | ☆☆☆ | ☆ | ☆☆☆ | 7/9 |
| Abdelsadek, A., et al., 2016 [26] | Cross-sectional | ☆☆ | - | - | 2/10 |
| Rachuri, H., et al., 2017 [27] | Cross-sectional | ☆☆☆☆☆ | ☆ | ☆☆ | 8/10 |
| Perri, A.; et al., 2018 [28] | Cohort | ☆☆☆☆ | - | ☆☆☆ | 7/9 |
| Li, Y.; Lin, L.; Wang, Q., 2018 [29] | Cross-sectional | ☆☆☆ | ☆☆ | ☆☆ | 7/10 |
| Grimaldi, C., et al., 2019 [30] | Cohort | ☆☆☆☆ | ☆ | ☆☆☆ | 8/9 |
| Pasic, I.S., et al., 2020 [31] | Cross-sectional | ☆☆☆ | ☆ | ☆☆ | 6/10 |
| Vardar, G., et al., 2020 [32] | Cohort | ☆☆☆ | - | ☆☆☆ | 6/9 |
| Kayki, G., et al., 2021 [33] | Cohort | ☆☆☆ | ☆ | ☆☆ | 6/9 |
| Aldecoa-Bilbao, V.; et al., 2021 [34] | Cohort | ☆☆☆☆ | ☆☆ | ☆☆☆ | 9/9 |
| Oktem, A., et al., 2021 [35] | Cohort | ☆☆ | - | ☆☆ | 4/9 |
| Reza, M.; et al., 2021 [36] | Cross-sectional | ☆☆ | - | ☆☆ | 4/10 |
| Eldeen, S.M.; et al., 2022 [37] | Case-control | ☆☆☆ | ☆☆ | ☆☆☆ | 8/9 |
| Xiao, Y., et al., 2022 [38] | Cohort | ☆☆☆ | ☆ | ☆☆ | 6/9 |

Score ranges to qualitatively categorize the overall quality of the cohort and case-control studies: 0 to 4=poor quality; 5 to 7=fair quality; 8 to 9=high quality. For cross-sectional studies: 0 to 4=Unsatisfactory quality; 5 to 6=Satisfactory quality; 7 to 8= good quality; 9 to 10=high quality [15]

**REFERENCES**

1. Walani SR. Global burden of preterm birth. Int J Gynecol Obstet. 2020;150(1):31–33. doi: 10.1002/ijgo.13195

2. Patel AB, Bann CM, Kolhe CS, Lokangaka A, Tshefu A, Bauserman M, et al. The Global Network Socioeconomic Status Index as a predictor of stillbirths, perinatal mortality, and neonatal mortality in rural communities in low and lower middle income country sites of the Global Network for Women’s and Children’s Health Research. Simeoni U, editor. PLOS ONE. 2022;17(8):e0272712. doi: 10.1371/journal.pone.0272712

3. Shukla VV, Eggleston B, Ambalavanan N, McClure EM, Mwenechanya M, Chomba E, et al. Predictive Modeling for Perinatal Mortality in Resource-Limited Settings. JAMA Netw Open. 2020;3(11):e2026750. doi: 10.1001/jamanetworkopen.2020.26750

4. Warren JB, Anderson JM. Core Concepts: Respiratory Distress Syndrome. NeoReviews. 2009;10(7):e351–e361. doi: 10.1542/neo.10-7-e351

5. Huang L, Ye D, Wang J. Analysis of diagnosing neonatal respiratory distress syndrome with lung ultrasound score. Pak J Med Sci. 2022 [cited 2023 Oct 21];[6 p.]. doi: 10.12669/pjms.38.5.5202

6. Rubarth LB, Quinn J. Respiratory Development and Respiratory Distress Syndrome. Neonatal Netw. 2015;34(4):231–238. doi: 10.1891/0730-0832.34.4.231

7. Reuter S, Moser C, Baack M. Respiratory Distress in the Newborn. Pediatr Rev. 2014;35(10):417–429. doi: 10.1542/pir.35.10.417

8. Laroia AT, Donnelly EF, Henry TS, Berry MF, Boiselle PM, Colletti PM, et al. ACR Appropriateness Criteria® Intensive Care Unit Patients. J Am Coll Radiol. 2021;18(5):S62–S72. doi: 10.1016/j.jacr.2021.01.017

9. Frija G, Blažić I, Frush DP, Hierath M, Kawooya M, Donoso-Bach L, et al. How to improve access to medical imaging in low- and middle-income countries? eClinicalMedicine. 2021;38:101034. doi: 10.1016/j.eclinm.2021.101034

10. Oza S, Cousens SN, Lawn JE. Estimation of daily risk of neonatal death, including the day of birth, in 186 countries in 2013: a vital-registration and modelling-based study. Lancet Glob Health. 2014;2(11):e635–e644. doi: 10.1016/S2214-109X(14)70309-2

11. Puchalski AL, Magill C. Imaging Gently. Emerg Med Clin North Am. 2018;36(2):349–368. doi: 10.1016/j.emc.2017.12.003

12. Page MJ, McKenzie JE, Bossuyt PM, Boutron I, Hoffmann TC, Mulrow CD, et al. The PRISMA 2020 statement: an updated guideline for reporting systematic reviews. BMJ. 2021;n71. doi:10.1136/bmj.n71.

13. Zamboni A, Hernandes E, Fabbri S. *StArt uma ferramenta computacional de apoio à revisão sistemática [StArt a computational tool to support systematic reviews].* Paper presented at: Congresso Brasileiro de Software (CBSoft’10); 2010; Salvador, BA, Brazil

14. Wells G, Shea B, O'Connell D, Peterson j, Welch V, Losos M, et al. The Newcastle–Ottawa Scale (NOS) for assessing the quality of nonrandomised studies in meta-analyses. Ottawa Health Research Institute; 2014.

15. Gierisch JM, Beadles C, Shapiro A, McDuffie JR, Cunningham N, Bradford D, et al. Health Disparities in Quality Indicators of Healthcare Among Adults with Mental Illness. Washington (DC): Department of Veterans Affairs (US); 2014. PMID: [26065051](https://pubmed.ncbi.nlm.nih.gov/26065051)

16. Wood BP, Sinkin RA, Kendig JW, Notter RH, Shapiro DL. Exogenous lung surfactant: effect on radiographic appearance in premature infants. Radiology. 1987;165(1):11–13. doi: 10.1148/radiology.165.1.3306780

17. Kurl S, Heinonen KM, Kiekara O. The First Chest Radiograph in Neonates Exhibiting Respiratory Distress at Birth. Clin Pediatr (Phila). 1997;36(5):285–289. doi: 10.1177/000992289703600506

18. Bober K, Swietliński J. Diagnostic utility of ultrasonography for respiratory distress syndrome in neonates. Med Sci Monit Int Med J Exp Clin Res. 2006;12(10):CR440-446. PMID: 17006405.

19. Shahramian I, Noori NM, Sharafi E, Ramezani AA, Hesaraki M. Brain Natriuretic Peptide: A Predictor for Severity Respiratory Distress Syndrome in Newborns. J Compr Pediatr. 2013;4(4):189–193. doi: 10.17795/compreped-11262

20. Raimondi F, Migliaro F, Sodano A, Ferrara T, Lama S, Vallone G, et al. Use of Neonatal Chest Ultrasound to Predict Noninvasive Ventilation Failure. Pediatrics. 2014;134(4):e1089–e1094. doi: 10.1542/peds.2013-3924

21. Yin X, Xie L, Chai Y, Fan H, Han X, Feng Z. Surfactant protein b expression in bronchoalveolar lavage fluid of full-term neonates with respiratory distress syndrome. Acta Clin Croat. 2014;53(2):161–165. PMID: 25163231.

22. Tagliaferro T, Bateman D, Ruzal-Shapiro C, Polin RA. Early radiologic evidence of severe respiratory distress syndrome as a predictor of nasal continuous positive airway pressure failure in extremely low birth weight newborns. J Perinatol. 2015;35(2):99–103. doi: 10.1038/jp.2014.164

23. El-Malah H, Hany S, Koriem M, Ali A. Lung ultrasonography in evaluation of neonatal respiratory distress syndrome. Egypt J Radiol Nucl Med. 2015;46(2):469–474. doi: 10.1016/j.ejrnm.2015.01.005

24. Liu J, Cao HY, Wang HW, Kong XY. The role of lung ultrasound in diagnosis of respiratory distress syndrome in newborn infants. Iran J Pediatr. Feb;25(1):e323. doi: 10.5812/ijp.323.

25. Sawires HK, Abdel Ghany EA, Hussein NF, Seif HM. Use of Lung Ultrasound in Detection of Complications of Respiratory Distress Syndrome. Ultrasound Med Biol. 2015;41(9):2319–2325. doi: 10.1016/j.ultrasmedbio.2015.04.02

26. Abdelsadek A, Khair MDA, Naga OA. Lung ultrasound as early diagnostic tool in neonatal respiratory distress syndrome (RDS). Egypt J Chest Dis Tuberc. 2016;65(1):377–382. doi: 10.1016/j.ejcdt.2015.07.006

27. Rachuri H, Oleti TP, Murki S, Subramanian S, Nethagani J. Diagnostic Performance of Point of Care Ultrasonography in Identifying the Etiology of Respiratory Distress in Neonates. Indian J Pediatr. 2017;84(4):267–270. doi: 10.1007/s12098-016-2288-7

28. Perri A, Riccardi R, Iannotta R, Di Molfetta DV, Arena R, Vento G, et al. Lung ultrasonography score versus chest X‐ray score to predict surfactant administration in newborns with respiratory distress syndrome. Pediatr Pulmonol. 2018;53(9):1231–1236. doi: 10.1002/ppul.24076

29. Li Y, Lin L, Wang Q. Correlation of expression levels of caspase-3 and Bcl-2 in alveolar lavage fluid in neonatal respiratory distress syndrome and prognosis. Exp Ther Med. 2018 [cited 2023 Oct 22]; [5 p.]. doi: 10.3892/etm.2018.5755

30. Grimaldi C, Michel F, Brévaut-Malaty V, Hassid S, Nicaise C, Puech B, et al. Thoracic ultrasound accuracy for the investigation of initial neonatal respiratory distress. Arch Pédiatrie. 2019;26(8):459–465. doi: 10.1016/j.arcped.2019.09.009

31. Sefic Pasic I, Terzic S, Nisandzic j, Pokrajac D. Lung ultrasound and neonatal respiratory distress syndrome. J Clin Neonatol. 2020;9(4):272. doi: 10.4103/jcn.JCN_69_20

32. Vardar G, Karadag N, Karatekin G. The Role of Lung Ultrasound as an Early Diagnostic Tool for Need of Surfactant Therapy in Preterm Infants with Respiratory Distress Syndrome. Am J Perinatol. 2021;38(14):1547–1556. doi: 10.1055/s-0040-1714207

33. Kayki G, Yigit S, Tandircioglu UA, Celik HT, Yurdakok M. Lung ultrasound (LUS) and surfactant treatment: looking for the best predictive moment. J Perinatol. 2021;41(7):1669–1674. doi: 10.1038/s41372-021-01039-0

34. Aldecoa-Bilbao V, Balcells-Esponera C, Herranz Barbero A, Borràs-Novell C, Izquierdo Renau M, Iriondo Sanz M, et al. Lung ultrasound for early surfactant treatment: Development and validation of a predictive model. Pediatr Pulmonol. 2021;56(2):433–441. doi: 10.1002/ppul.25216

35. Oktem A, Yigit S, Oğuz B, Celik T, Haliloğlu M, Yurdakok M. Accuracy of lung ultrasonography in the diagnosis of respiratory distress syndrome in newborns. J Matern Fetal Neonatal Med. 2021;34(2):281–286. doi: 10.1080/14767058.2019.1605350

36. Reza M, Utomo M, Aden T. Comparison between lung ultrasonography and chest x-ray in the diagnosis of respiratory distress syndrome in preterm neonates in Dr. Soetomo General Hospital Surabaya, Indonesia. Curr Pediatr Res; 2021,25(4): 570-574. ISSN 0971-9032

37. Mohy Eldeen S, Ali S, Salama H. Clinical characteristics, diagnosis, and management outcome of surfactant deficiency respiratory distress syndrome in term and near-term neonates. A retrospective observational study. Acta Biomed Atenei Parm. 2022;93(6):e2022337. doi: 10.23750/abm.v93i6.13794

38. Xiao Y, Guo, Ting. CLINICAL VALUE OF LUNG ULTRASOUND IN THE DIAGNOSIS AND FOLLOW-UP TREATMENT OF NRDS. Acta Medica Mediterr. 2022;3:1911–1918. doi: 10.19193/0393-6384_2022_3_293

39. Hiles M, Culpan AM, Watts C, Munyombwe T, Wolstenhulme S. Neonatal respiratory distress syndrome: Chest X-ray or lung ultrasound? A systematic review. Ultrasound. 2017;25(2):80–91. doi: 10.1177/1742271X16689374

40. Sweet DG, Carnielli VP, Greisen G, Hallman M, Klebermass-Schrehof K, Ozek E, et al. European Consensus Guidelines on the Management of Respiratory Distress Syndrome: 2022 Update. Neonatology. 2023;120(1):3–23. doi: 10.1159/000528914

41. Ho JJ, Subramaniam P, Davis PG. Continuous positive airway pressure (CPAP) for respiratory distress in preterm infants. Cochrane Database Syst Rev. 2020 [cited 2023 Oct 22]; [43 p.]. doi: 10.1002/14651858.CD002271.pub3
